# 80例晚期NSCLC吉非替尼治疗长于6个月患者的预后因素分析

**DOI:** 10.3779/j.issn.1009-3419.2010.11.10

**Published:** 2010-11-20

**Authors:** 玲 戴, 健 方, 鋆 聂, 维亨 胡, 筱玲 陈, 金娣 韩, 广明 田, 森 韩, 叙仪 刘

**Affiliations:** 100142 北京，北京大学肿瘤医院暨北京市肿瘤防治研究所，恶性肿瘤发病机制及转化研究教育部重点实验室 Key Laboratory of Carcinogenesis and Translational Research (Ministry of Education), Peking University Cancer Hospital and Institute, Beijing 100142, China

**Keywords:** 肺肿瘤, 吉非替尼, 靶向治疗, Lung neoplasms, Gefitinib, Targeted therapy

## Abstract

**背景与目的:**

表皮生长因子受体酪氨酸激酶抑制剂（epidermal growth factor receptor tyrosine kinase inhibitor, EGFR-TKI）治疗非小细胞肺癌（non-small cell lung cancer, NSCLC）有效的临床预测指标包括：女性、亚裔、无吸烟史、腺癌类型以及产生皮疹等，本研究的目的是探寻吉非替尼治疗无进展生存期（progression-free survival, PFS）≥6个月的NSCLC患者的临床预后因素。

**方法:**

2005年1月-2010年3月我组经治80例吉非替尼治疗PFS≥6个月的NSCLC病例，分析其临床特点与PFS的相关性。

**结果:**

年龄 > 70岁、更早的分期（Ⅲb）、非骨转移患者显示出更长的中位PFS（27个月*vs* 12个月；32个月*vs* 12个月；16个月*vs* 10个月，*P* < 0.05）。ECOG-PS评分0分-1分较2分-3分者、既往化疗周期数多于4周期或化疗PFS长于6个月者较化疗不足4周期或缓解≤6个月者的吉非替尼中位PFS似乎更长（15个月*vs* 10个月；16个月*vs* 12个月；14个月*vs* 12个月），但差异无统计学意义（*P* > 0.05）。服药后出现皮疹及皮疹Ⅱ度以上者的中位PFS较无皮疹或皮疹0度-Ⅰ度者更长（16个月*vs* 13个月，*P*=0.17；19个月*vs* 11个月，*P*=0.085），但差异无统计学意义。性别、吸烟指数、病理类型、除骨以外的转移部位、初治或复治等因素与吉非替尼治疗长期获益患者的PFS无关（*P* > 0.05）。

**结论:**

年龄 > 70岁、更早的分期（Ⅲb）岁、非骨转移患者进行吉非替尼治疗可能获得较长的PFS。

## Abstract (en)

**Background and objective:**

Some clinical predictors can be used to evaluate the efficacy of epidermal growth factor receptor tyrosine kinase inhibitor (EGFR-TKI) therapy for non-small cell lung cancer (NSCLC), including female, East-Asian, non-smoker, adenocarcinoma, skin rash, *etc*. The aim of this study is to explore the prognosis of advanced NSCLC patients treated with gefitinib for more than 6 months.

**Methods:**

Eighty advanced NSCLC patients treated with gefitinib for more than 6 months were collected from January, 2005 to March, 2010. The association of their clinical characteristics with median progression-free survival time (PFS) was analysed.

**Results:**

Significantly longer median PFS were found in patients with > 70 years old, earlier clinical stage (Ⅲb), non-bone metastasis (27 months *vs* 12 months; 32 months *vs* 12 months; 16 months *vs* 10 months, *P* < 0.05). There was no significant difference in median PFS between ECOG performance status 0-1 group and 2-4 group, between more than 4 cycles of chemotherapy and 1-4 cycles, between PFS of chemotherapy > 6 months group and ≤6 months group, however, ECOG 0-1 group and more than 4 cycles of chemotherapy or PFS of chemotherapy > 6 months group seemed to have longer median PFS (15 months *vs* 10 months; 16 months *vs* 12 months; 14months *vs* 12 months). Compared with no skin rash and grade 0-Ⅰ rash group, the patients with rash or grade ≥Ⅱ rash had longer median PFS (16 months *vs* 13 months, *P*=0.171; 19 months *vs* 11 months, *P*=0.085). The median PFS was not related with sex, smoking index, pathological types, metastatic sites except bone, treatment strategy, *etc* (*P* > 0.05).

**Conclusion:**

For gefitinib treatment, longer median PFS is likely to be obtained in patients with > 70 years old, earlier clinical stage (Ⅲb), non-bone metastasis.

肺癌是癌症患者死亡的第一位病因^[1]^，其中非小细胞肺癌（non-small cell lung cancer, NSCLC）占肺癌的80%以上^[2]^。大多数患者在诊断时即为不可切除的晚期肺癌^[3]^。目前传统化疗疗效已进入了一个平台期，靶向治疗的出现使晚期非小细胞肺癌的治疗有了更多选择，其中小分子表皮生长因子受体酪氨酸激酶抑制剂（epidermal growth factor receptor tyrosine kinase inhibitor, EGFRTKI）的临床研究进展备受瞩目。

吉非替尼（gefitinib）是EGFR-TKI的代表药物之一^[4, 5]^。多项临床试验^[6-8]^结果表明EGFR-TKI治疗晚期NSCLC有效的临床预测指标包括：女性、亚裔、无吸烟史、腺癌类型以及产生皮疹等。本文通过分析服吉非替尼长期生存患者[无进展生存期（progression-free survival, PFS）≥6个月]的临床特点探寻这类患者可能的预后因子，为临床筛选吉非替尼治疗长期生存受益患者提供帮助。

## 1 材料与方法

### 1.1 病例资料

2005年1月-2010年3月间连续收治的80例经组织学或细胞学确诊的NSCLC患者，全组患者吉非替尼治疗的疾病控制期≥6个月。男性20例，女性60例。中位年龄65岁（40岁-85岁）。全组分期均为Ⅲb期-Ⅳ期（1997年分期），其中，Ⅲb期15例，Ⅳ期65例。吉非替尼作为二线及以上治疗者56例，一线治疗者24例。

### 1.2 治疗方法

口服吉非替尼250 mg/d，至疾病进展或毒副反应不能耐受。服药同时进行局部治疗者15例（肺内局部病灶治疗含胸腔化疗7例，骨转移放疗3例，脑转移放疗3例，锁骨上淋巴结放疗1例，胸壁种植转移灶切除1例）。

### 1.3 评价与随访

吉非替尼治疗期间每2-3个月随访1次。随访时按WHO标准评价疗效及药物毒副反应。疗效评定分为：完全缓解（complete response, CR），部分缓解（partial response, PR），稳定（stable diease, SD），进展（progressive diease, PD），CR+PR+SD为疾病控制率（diease control rate, DCR）；总生存期（overall survival, OS）：开始治疗至死亡或末次随访时间。PFS：开始治疗至肿瘤复发或进展的时间。毒副反应以0度-Ⅳ度进行评价。全组病例随访期：6个月-60个月（中位随访期：16个月），随访期内52例发生疾病进展，截止2010年3月尚有28例患者在接受吉非替尼治疗。失访3例。

### 1.4 统计学处理

数据统计学处理采用SPSS 17.0统计软件进行分析。*Kaplan-Meier*生存分析采用*Log-rank*检验，以*P* < 0.05为有统计学差异。

## 2 结果

### 2.1 临床特点统计

患者临床特点与PFS之间的关系见表 1，患者治疗特点与PFS之间的关系见表 2。

**1:** 患者临床特点与PFS之间的关系 Relationship between PFS and clinical characteristics

	*n*	Rate (%)	Median PFS (month)	*P*
Gender				0.939
Male	20	25.00	11	
Female	60	75.00	14	
Age (years)				0.027
> 70	24	30.00	27	
≤70	56	70.00	12	
Smoking index				0.921
≥10	11	13.75	11	
< 10	69	86.25	14	
ECOG PS				0.165
≤1	65	81.25	15	
≥2	15	18.75	10	
Histology				0.984
Adenocarcinoma	69	86.25	14	
Non-adenocarcinoma	11	13.75	14	
TNM stage^*^				0.038
Ⅳ	65	82.28	12	
Ⅲb	14	17.72	32	
Site of metastasis				
Intrapulmonary metastasis	56	70.00	14	0.862
Non-intrapulmonary metastasis	24	30.00	15	
Liver metastasis	7	8.75	17	0.388
Non-liver metastasis	73	91.25	13	
Brain metastasis	26	32.50	12	0.135
Non-brain metastasis	54	67.50	15	
Bone metastasis	34	42.50	10	0.013
Non-bone metastasis	46	57.50	16	
^*^: The TNM stage of 1 patient was uncertain before the initial gefitinib treatment. PFS: progression-free survival.				

**2:** 患者治疗特点与PFS之间的关系 Relationship between PFS and therapeutic characteristics

	*n*	Rate (%)	Median PFS (month)	*P*
Treatment strategy				0.540
1^st^ line	24	30.00	15	
≥2^nd^ line	56	70.00	13	
Previous chemotherapy^◎^				0.082
> 4 cycles	30	48.39	16	
≤4 cycles	32	51.61	12	
Previous chemotherapy PFS^◎^				0.252
> 6 months	32	51.61	14	
≤6 months	30	48.39	12	
Skin rash				0.171
No	50	62.50	13	
Yes	30	37.50	16	
Grade 0-1 rash	57	71.25	11	0.085
Grade 2-4 rash	23	28.75	19	
Grade 0-1 diarrhea	69	86.25	12	0.861
Grade 2-4 diarrhea	11	13.75	19	
◎：Sixty-two patients had received chemotherapy before gefitinib treatment, including neo-adjuvant chemotherapy, adjuvant chemotherapy and salvage chemotherapy.				

### 2.2 PFS及生存统计分析

80例患者的中位PFS为14个月，中位OS为34个月（95%CI: 25.4-42.6），1年生存率为83.4%（67/80）（图 1）。

**1:**
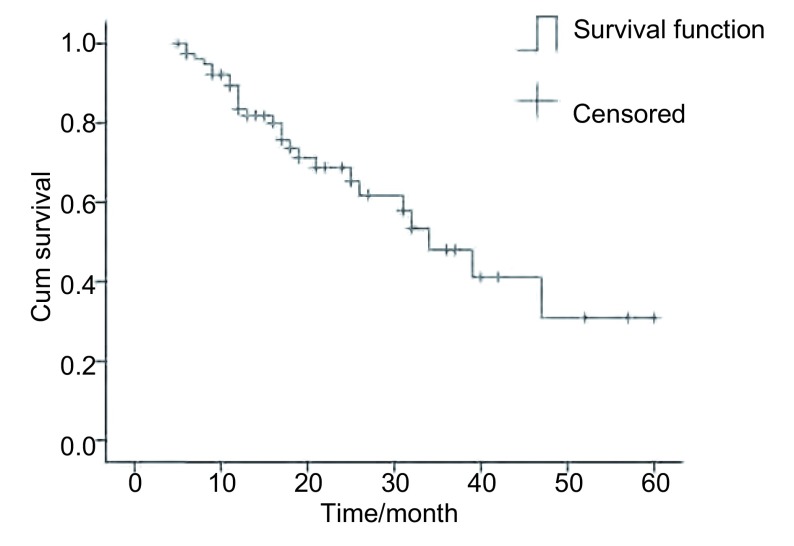
80例NSCLC患者生存曲线 *Kaplan-Meier* survival curve of the 80 patients with NSCLC

>70岁患者较≤70岁者有更长的中位PFS（27个月*vs* 12个月，*P*=0.027）；Ⅲb期组较Ⅳ期组也具有更长的中位PFS（32个月*vs* 12个月，*P*=0.038）。服药时患者体能状况评分（ECOG performance status, PS）0分-1分者中位PFS长于ECOG≥2分者，但差异无统计学意义（15个月*vs* 10个月，*P*=0.165）（图 2）。

**2:**
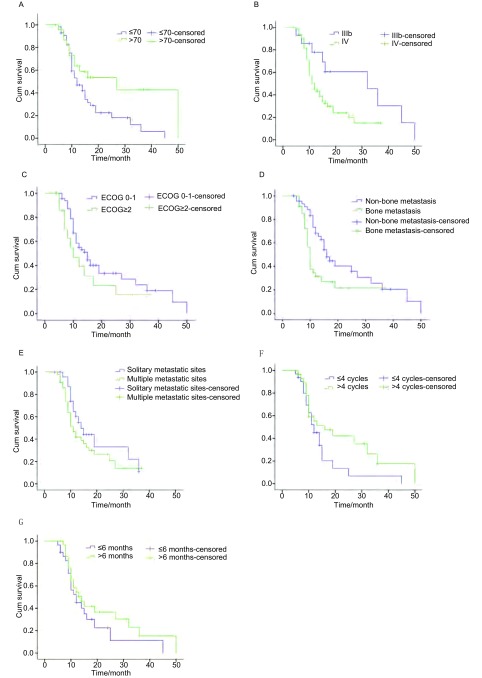
80例晚期NSCLC患者不同变量的PFS曲线。A：年龄；B：TNM分期；C：体能状况评分；D：骨转移与非骨转移；E：单一部位转移与多部位转移；F：既往化疗周期数；G：既往化疗PFS。 Progression free survival curves of different variables of the 80 patients with advanced NSCLC. A: Age; B: TNM stage; C: ECOG performance status; D: Bone metastasis and non-bone metastasis; E: Solitary and multiple metastatic sites; F: The number of cycles of previous chemotherapy; G: PFS of previous chemotherapy.

无骨转移者的中位PFS要长于骨转移者（16个月*vs* 10个月，*P*=0.013）。但单一部位转移患者的中位PFS似乎要长于多部位转移者，但差异无统计学意义（14个月*vs* 11个月，*P*=0.071）（图 2）。

62例既往接受过化疗的患者中，化疗4周期以上或化疗PFS>6个月者的中位PFS略长于化疗周期≤4，化疗PFS≤6个月者，但差异无统计学意义（16个月*vs* 12个月，14个月*vs* 12个月，*P*>0.05）（图 2）。

## 3 讨论

吉非替尼属于EGFR-TKI，通过与表皮生长因子受体（epidermal growth factor receptor, EGFR）特异性结合，阻断信号传导，抑制肿瘤细胞增殖、侵袭、浸润，促其凋亡，延长NSCLC患者的生存期^[9]^。临床试验提示EGFRTKI治疗有效的临床预测指标包括：女性、亚裔、无吸烟史、腺癌类型以及产生皮疹等^[4-6]^。本研究80例服用吉非替尼获得超过6个月疾病控制的病例中亦同样发现女性、少吸烟（< 10包年）、腺癌的患者比例占多数，分别为75%、86.25%、86.25%，进一步分析发现：此长期获益人群中不同性别、吸烟指数及病理类型患者的PFS未显示差异，即吉非替尼治疗敏感的男性、吸烟、非腺癌患者也可获得较长的疾病控制期。2010年ASCO发表的一项1 698例接受吉非替尼治疗的晚期NSCLC研究也有类似提示：130例吉非替尼治疗后缓解率（response rate, RR）或SD≥6个月患者的PFS与年龄、性别、组织学类型、吸烟状态及吉非替尼给药时机无关^[10]^。EGFR-TKI治疗的临床预测指标是否就是治疗预后指标有待更多临床数据验证。

IPASS等研究^[11, 12]^显示老年人群具有更高的EGFR突变比例，提示老年人群是吉非替尼治疗的优势人群。本研究发现年龄>70岁患者的中位PFS为27个月，明显好于年龄≤70岁者的12个月（*P*=0.027），这一结果提示：对于吉非替尼治疗有效的患者，年龄可能是其能否长期获益的预后因素之一。

肿瘤分期和PS评分均是NSCLC患者的最重要的预后因素^[13-15]^。本研究中Ⅳ期患者占82.28%（65/79）、具有2个部位以上转移者占55.70%（44/79），与临床应用吉非替尼的实际情况相似。但进一步分析PFS曲线（图 2）发现：更早的分期（Ⅲb期）、更少的转移部位（单一部位）具有更长的中位PFS（32个月*vs* 12个月，*P*=0.038；14个月*vs* 11个月，*P*=0.071）。这可能与这类患者的瘤负荷更小、受影响的脏器更少有关。与分期影响预后的原因相似，PS评分好的患者具有更好的脏器功能和治疗耐受性，是肿瘤预后良好的因素之一。本研究中PS评分0分-1分的患者中位PFS长于2分-4分者，为15个月*vs* 10个月，同样提示PS评分好的患者较评分差者更能从吉非替尼治疗中长期获益。由于病例数不足的原因，差异无统计学意义（*P*=0.165），应进一步扩大样本量观察。

肺、骨、脑、肝是临床上NSCLC常见的转移部位，本研究80例患者中肺、骨、脑、肝转移的发生率分别为70%、42.5%、32.5%、8.75%，不同转移部位对吉非替尼的远期疗效影响不尽相同。吉非替尼治疗长期获益的人群中存在肺转移的患者比例明显高于其它部位，且肺转移患者的中位PFS并不差于无肺转移者（14个月*vs* 15个月，*P*=0.862），似乎提示肺转移并未影响吉非替尼治疗的远期疗效，并非预后不良的因素。无骨、脑转移患者的中位PFS均较有转移者延长（16个月*vs* 10个月，*P*=0.013；15个月*vs* 12个月，*P*=0.135），提示无骨、脑转移者经过吉非替尼治疗可能会获得更长的疾病控制期，但由于病例数有限，本研究未再对伴或不伴症状的骨、脑转移进行分层分析，因此该结果应继续扩大样本量进行验证。肝转移被认为是NSCLC的预后不良因素之一，本研究中肝转移患者仅有7例（8.75%），由于病例数较少，统计学分析未显示出肝转移对PFS的影响，但在本研究80例吉非替尼治疗长期获益的人群中存在肝转移的患者比例明显少于其它常见转移部位，似乎也说明肝转移患者的远期疗效不佳。

本研究中初治患者24例（30%），中位PFS为15个月（95%CI: 9.2-20.8），中位生存期为32个月（95%CI: 18.5-45.5）；复治者56例（70%），中位PFS为13个月（95%CI: 8.8-17.2），中位生存期为47个月（95%CI: 23.6-54.4），较初治者明显延长。由于病例数较少，两组在PFS及OS方面的差异无统计学意义（*P*=0.540, *P*=0.936）。由于检测条件所限，本研究并未要求检测EGFR基因突变，属于TKI治疗的非选择人群，所得结果与2010年ASCO发表的台湾类似研究结果相似^[16]^。说明对于吉非替尼治疗有效的患者，其能否获得长期的疾病控制与是否为初治无关。

对于复治患者，既往化疗的疗程及疗效可能影响吉非替尼治疗的远期疗效。本研究结果显示：既往化疗4个周期以上或化疗的PFS>6个月者的中位PFS长于既往化疗1个-4个周期或化疗PFS≤6个月者（16个月*vs* 12个月，*P*=0.082；14个月*vs* 12个月，*P*=0.252），从PFS曲线上也可见此趋势（图 2）。这种情况的发生可能与既往化疗周期数多、PFS长的患者具有某些好的预后因素有关，使得这类患者对化疗和靶向治疗都有好的疗效。本研究尚未发现这类因素，但这一结果却提示：既往接受多周期化疗或化疗的PFS较长的患者换用吉非替尼治疗时可能获得较好的远期疗效。

皮疹被看作吉非替尼或厄洛替尼治疗有效的预测指标之一^[4-6]^。本研究80例患者中出现皮疹者的中位PFS达16个月，Ⅱ度及以上皮疹者的中位PFS更达到19个月，较无皮疹者的13个月（*P*=0.171）和0度-Ⅰ度皮疹者的11个月（*P*=0.085）明显延长，提示皮疹的出现和程度可能是吉非替尼治疗的预后因素。而另一个吉非替尼治疗常见的副反应腹泻则未发现类似情况。

## 4 结论

对于吉非替尼治疗有效的患者来说，年龄>70岁、更早的分期（Ⅲb）、无骨转移的患者可能获得更长的PFS，而一般状况好、无脑转移、既往化疗多周期及PFS长、治疗中出现严重皮疹等都有可能是获得好的远期疗效的有利因素，值得进一步扩大样本量并结合分子生物学基因检测进行研究验证。

## References

[1] Parkin DM, Bray F, Ferlay J (2005). Global cancer statistics. 2002. CA Cancer J Clin.

[2] Govindan R, Page N, Morgensztern D (2006). Changing epidemiology of small-cell lung cancer in the United States over the last 30 years: Analysis of the surveillance, epidemiologic, and end results database. J Clin Oncol.

[3] Spira A, Ettinger DS (2004). Multidisciplinary management of lung cancer. N Engl J Med.

[4] Reck M (2009). Gefitinib in the treatment of advanced non-small-cell lung cancer. Expert Rev Anticancer Ther.

[5] Jiang HY (2009). Overview of gefitinib in non-small-cell lung cancer: an Asian perspective. Jpn J Clin Oncol.

[6] Thatcher N, Chang A, Parikh P (2005). Gefitinib plus best supportive care in previously treated patients with refractory advanced non-small-cell lung cancer: Results from a randomized, placebo controlled, multicentre study(Iressa Survival Evaluation in Lung Cancer). Lancet.

[7] Shepherd FA, Rodrigues PJ, Ciuleanu T (2005). Erlotinib in previously treated non-small-cell lung cancer. N Engl J Med.

[8] West HL, Franklin WA, McCoy J (2006). Gefitinib therapy in advanced bronchioloalveolar carcinoma: Southwest Oncology Group Study S0126. J Clin Oncol.

[9] Wang Y, Xu JM, Song ST (2005). Research progresses on mechanisms of epidermal growth factor receptor targeted drugs and their related markers. Chin J Oncol.

[b10] 10Lee Y, Yun T, Han J, *et al*. Survival predictors in advanced non-small cell lung cancer (NSCLC) patients with clinically proven sensitivity to gefitinib. J Clin Oncol 28, 2010 (suppl; abstr e18060).

[b11] 11Fukuoka M, Wu Y, Thongprasert S, *et al*. Biomarker analyses from a phase Ⅲ, randomized, open-label, first-line study of gefitinib(G) versus carboplatin/ paclitaxel(C/P) in clinically selected patients(pts) with advanced nonsmall cell lung cancer(NSCLC) in Asia(IPASS). J Clin Oncol, 27: 15s, 2009 (suppl; abstr 8006).

[12] Rosell R, Moran T, Queralt C (2009). Screening for epidermal growth factor receptor mutations in lung cancer. N Engl J Med.

[13] Brundage MD, Davies D, Mackillop WJ (2002). Prognostic factors in non-small cell lung cancer: A decade of progress. Chest.

[14] Mountain CF (1997). Revisions in the international system for staging lung cancer. Chest.

[15] Takigawa N, Segawa Y, Okahara M (1996). Prognostic factors for patients with advanced non-small cell lung cancer: univariate and multivariate analyses including recursive partitioning and amalgamation. Lung Cancer.

[b16] 16Lie C, Lin M, Chao T, *et al*. First- or second-line gefitinib therapy in unknown epidermal growth factor receptor mutants of non-small cell lung cancer patients. J Clin Oncol, 28: 15s, 2010 (suppl; abstr 7577).

